# Exceptionally High Negative Electro-Caloric Effects of Poly(VDF–*co*–TrFE) Based Nanocomposites Tuned by the Geometries of Barium Titanate Nanofillers

**DOI:** 10.3390/polym9080315

**Published:** 2017-07-28

**Authors:** Zhi-Yuan Jiang, Guang-Ping Zheng, Xiu-Cheng Zheng, Hao Wang

**Affiliations:** 1 Department of Mechanical Engineering, Hong Kong Polytechnic University, Hung Hom, Kowloon, Hong Kong, China; jiang.zhiyuan@connect.polyu.hk; 2 College of Chemistry and Molecular Engineering, Zhengzhou University, Zhengzhou 450001, China; 3 Institute of Nanosurface Science and Engineering, Shenzhen University, Shenzhen 518060, China

**Keywords:** ferroelectric polymers, phase transition, electro-caloric effect, nanofillers, nanocomposites, dielectric properties, mechanical relaxation

## Abstract

Exceptionally high electro-caloric effects (ECEs) are observed in nanocomposites consisting of poly(vinylidene fluoride-*co*-trifluoroethylene) (VDF–*co*–TrFE) copolymer and barium titanate (BT) nanoparticles and nanowires. The poly(VDF–*co*–TrFE) matrix nanocomposites containing 5% volume fraction of BT nanowires are found to exhibit a negative ECE temperature change as large as 12 °C or a refrigeration effect of 8.3 J/g, which is much larger than those reported to date. The mechanisms of negative ECE and the enhanced negative ECE in the nanocomposites consisting of poly(VDF–*co*–TrFE) and BT nanowires are explained by the Kauzmann theory on glassy polar states and the interaction between BT nanofillers and the copolymer matrix. The effects of geometries of BT nanofillers on the negative ECEs are elucidated by P-E loop measurements, and dielectric and dynamical mechanical analyses. The nanocomposites, with their enhanced negative ECE tuned by the geometries of BT nanofillers, provide us with promising ECE refrigerants for practical application to small-sized and environmentally-friendly ECE coolers in the heat management of electronic devices.

## 1. Introduction

The electro-caloric effect (ECE) of ferroelectrics has been studied for decades since it was first discovered by Kobeko and Kurchatov in 1930 [1]. Because the ECE refrigerants are small and efficient in a refrigeration cycle, the ECE of ferroelectrics is very promising for solid-state refrigeration, especially in the cooling of compact and mobile electronics with high power and energy densities. During the adiabatic polarization process, the dipoles in a ferroelectric material are arranged along an applied electric field and tend to be ordered. Consequently, the entropy of the ferroelectric material decreases, and heat is produced and the temperature increases under the adiabatic condition. With the removal of the electric field, the dipoles become relatively disordered, resulting in an increase of entropy of the materials [2]. As a result, the refrigeration effect of ferroelectrics is achieved. Although giant ECEs have been found in ferroelectric polymers, ceramics and polymer-ceramic composites, much effort is still devoted to developing those novel electronic materials which could possess large ECE entropy change [3,4,5,6,7,8,9]. Remarkably, some ferroelectric materials exhibit abnormal ECE which is in contrast to those aforementioned, suggesting that cooling occurs in the ECE materials with the application of an electric field and heating occurs in the materials with its removal. Abnormal ECE was observed from both direct and indirect measurements [10,11,12,13,14,15,16]. It has been found that the negative ECE temperature changes could be as high as ~6 °C in (Pb_0.97_La_0.02_)(Zr_0.95_Ti_0.05_)O_3_ thin films and ~1 °C in PbZrO_3_ thin films, whereas they decrease with increasing applied fields [14,15]. Negative ECEs have been also found in some lead-free ferroelectric ceramics, which could be more environmentally friendly when they are utilized as solid-state coolers for electronic devices [9,10]. However, the negative ECE temperature changes of those lead-free ferroelectrics are typically small (<6 °C), which have prevented them from such a practical application.

In this work, we report the negative ECE in ferroelectric copolymer-ceramic nanocomposite with the absolute value of ECE temperature change larger than 12 °C, which is much larger than those reported to date. The nanocomposite consists of poly(VDF–*co*–TrFE) copolymer [17] and barium titanate (BT) nanoparticles and nanowires with typical sizes smaller than 30 nm. It is found that the BT nanowires in the nanocomposites are more effective in enhancing the negative ECE of poly(VDF–*co*–TrFE) copolymer than the BT nanoparticles. The effects of BT nanofillers on the polar states of poly(VDF–*co*–TrFE) copolymer can be elucidated by dielectric analysis, and P-E loop and dynamical mechanical relaxation measurements. The negative ECE in the nanocomposite is further explained by the Kauzmann theory for ferroelectrics in a glassy polar state, which is a novel mechanism well describing the negative ECE tuned by the geometry and content of nanofillers.

## 2. Materials and Methods 

BaTiO_3_ nanowires (BTNWs) were prepared by a one-step hydrothermal route. In brief, polyethylene glycol (PEG-6000, 4 g) was dissolved into absolute ethanol (56 mL) in a 200 mL Teflon-lined stainless-steel autoclave. Subsequently, a solution of absolute ethanol (40 mL) and tetrabutyl titanate (1.3614 g, 4 mmol), a KOH alkaline solution (48 mL, 2 mol·L^−1^) of ethanol and deionized water (1:1) were added, respectively. The mixture was stirred for 0.5 h. Then Ba(OH)_2_·8H_2_O (1.2619 g, 4 mmol) was added and the mixture continued to be stirred for another 0.5 h. The autoclave was heated at 200 °C for 12 h. The pH value of the mixture was adjusted to about 4.0 by formic acid solution (6 mol·L^−1^). The mixture along with deionized water was then centrifuged at 10,500 rpm until its pH value was about 7.0. Solids were obtained from the mixture after centrifugation. Finally, the as-prepared solids were centrifuged with ethanol twice and were dried in air at 100 °C overnight, and BaTiO_3_ nanowires were obtained. The BaTiO_3_ nanoparticles (BTNPs) were also fabricated by a hydrothermal method. The barium chloride (BaCl_2_·2H_2_O) was dissolved in the deionized water. Based on an initial precursor molar ratio Ba/Ti of 1.6, the hydrochloric acid (HCl) solution of 15.0% titanium trichloride (TiCl_3_) was mixed with the solution of BaCl_2_. The pH value of the solution was adjusted to 13.5 with KOH solution (10 mol·L^−1^). After stirring for 1.5 h, the solution was transferred to an autoclave and held at 150 °C for 8 h to crystallize the reactants. When the solution was cooled to room temperature, the pH value of the final solution was adjusted to 6. The expected BaTiO_3_ powders were obtained after careful washing and drying at 105 °C.

The BaTiO_3_ nanowires or nanoparticles were dispersed in DMF by sonification for 30 mins. Then the powders of poly(VDF–*co*–TrFE) copolymer (P(VDF–TrFE), molar ratio of VDF:TrFE = 52:48, Piezotech, France) were added slowly to the solution under magnetic stirring. The solution was stirred for 8 h at 75 °C, and the obtained viscous solution was cast on copper foil with a thickness of 10–15 μm by a special blade. To remove the DMF, the film was then put into a vacuum oven for 8 h, resulting in P(VDF–TrFE)–BT nanocomopsites containing P(VDF–TrFE) matrix and BT nanofillers. Finally, the sample was held at 120 °C for 8 h for the crystallization of P(VDF–TrFE) in the nanocomposite films. The silver paste was coated on the top surface of these thick films to form an electrode while the other electrode was the copper substrate. For comparison of electrocaloric properties with those of thin-film samples, the above-mentioned solution was spin-coated onto an ITO substrate to form a nanocomposite thin film with a thickness of 87 nm. Graphene oxides dissolved in DMF were spin-coated at the top surface of the thin film, and then heat-treated at 120 °C. Consequently, a graphene (reduced graphene oxide) electrode with a thickness of 150 nm was formed at the top surface of the nanocomposite thin film.

The phases of nanocomposites were investigated by XRD (Rigaku Smartlab with Cu Kα radiation, λ = 0.15418 nm, Tokyo, Japan) which was operated at a tube voltage of 45 kV and a current of 200 mA. Raman spectra were obtained from a HR-800 Raman spectrometer with an argon ion laser emitted at a wavelength of 488 nm. Functional groups in the nanocomposites were determined by a Fourier transform infra-red (FT-IR) spectrometer (Vertex 70, Bruker Corp, Billerica, MA, USA) with the KBr dilution technique. The morphology of samples was determined by scanning electron microscopy (SEM) (Hitachi 4800, Tokyo, Japan) with an accelerating voltage of 5 kV. Transmission electron microscopy (TEM) images were taken with a FEI TGF 30 transmission electron microscope (Hillsboro, OR, USA) operated at an accelerating voltage of 200 kV. For TEM analysis, powders of BT nanowires were treated by strong sonication in ethanol and then dispersed onto a copper grid. Ferroelectric hysteresis loops were measured by a ferroelectric test system TF2000 (aixACCT, Aachen, Germany). The temperature dependent permittivity and dielectric loss were characterized by the impedance analyzer (HP 4192A, Washington, MA, USA). A dynamic mechanical analyzer (TA Instruments, DMA Q800, New Castle, DE, USA) was used to examine the mechanical relaxation processes of the samples.

## 3. Results

### 3.1. Characterizations on the P(VDF–TrFE)–BT Nanocomposites

**Figure 1:**
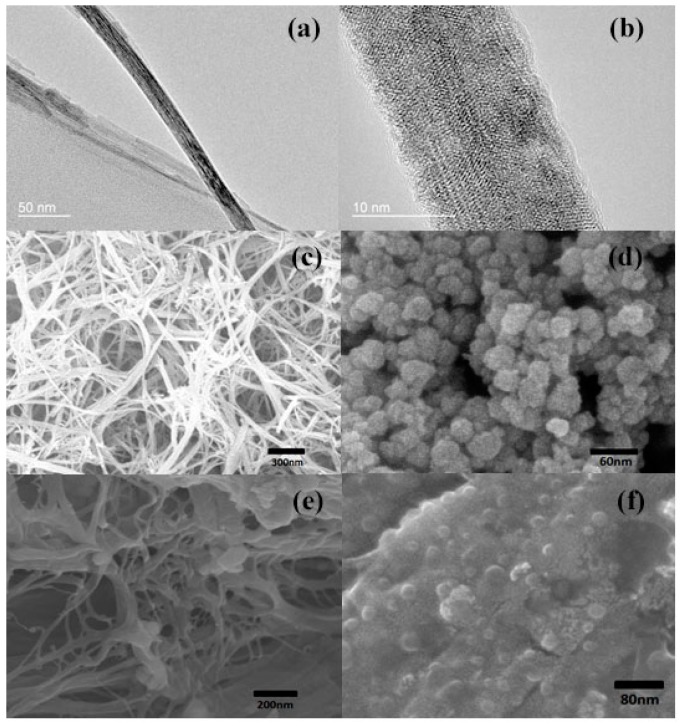
The TEM images of BT nanowires (**a**,**b**); SEM micrographs of powders of BT nanowires (**c**); and BT nanoparticles (**d**); Cross sections of P(VDF–TrFE)–BTNW nanocomposites with 10% volume fraction of BT nanowires (**e**); and 10% volume fraction of BT nanoparticles (**f**).

The BT nanowires prepared by the one-step hydrothermal reaction exhibit an ultra-high aspect ratio. The diameter of the nanowires is as small as 20 nm, as shown in the TEM (Figure 1a,b) and SEM images (Figure 1c), which are much smaller than those of the nanowires (200–500 nm) prepared by the two-step hydrothermal method [18]. SEM measurements on powders of BT nanowires and nanoparticles can be also used to characterize their sizes, as shown in Figure 1c,d. The corresponding ultra-large specific surface area of BT nanowires might lead to strong interaction between copolymer and nanowires, alternating the ferroelectric states of surrounding long-ordered molecule chains in the copolymer matrix. The nanoparticles can be found to be as small as 20 nm, as shown in Figure 1d. Figure 1e demonstrates the existence of BT nanowires in the nanocomposites, in which the ultrathin nanowires are well distributed. The uniformly embedded BT nanoparticles in the copolymer matrix can be also distinguished in the nanocomposite, as shown in Figure 1f.

The X-ray diffraction (XRD) patterns of BT nanowires are shown in Figure S1a, which are in good agreement with those reported in previous studies [19]. The XRD patterns of copolymer P(VDF–TrFE) and the corresponding P(VDF–TrFE)–BTNW nanocomposites are also shown for comparison. As second-phase fillers, BT nanowires are not likely to affect the local compositions of VDF and TrFE in the copolymer matrix. Hence the typical XRD peaks for P(VDF–TrFE) and BT can be distinguished in the XRD patterns of the nanocomposites. However, a change of peak position in the range of 18° to 20° is observed, as shown in Figure S1b. The broad peak around 19° indicates the coexistence of crystalline non-polar α and polar β (all-trans) phases of the copolymer, corresponding to the diffractions of {020} planes at 18.7° and {110} planes at 19.2° for P(VDF–TrFE), respectively. The filling of BT nanowires in the P(VDF–TrFE) matrix obviously weakens the polar β phases, manifested by the (110) peak which shifts from its original position at 19.2° to 19°, as shown in Figure S1b. It is assumed that the local electric fields induced by the entangled and ultra-thin nanowires may affect the spatial configuration of long molecule chains of copolymer, resulting in changes in the polar state of P(VDF–TrFE) and associated crystalline structures. However, it seems that BT nanoparticles embedded in the P(VDF–TrFE) matrix do not alternate the polar β phases, as can be observed from the XRD patterns shown in Figure S1c. Figure S2 shows the FT-IR spectra of pristine copolymer and nanocomposites with different volume fractions of BT nanowires and nanoparticles. The three intense bands at 1288, 850, and 1400 cm^-1^ associated with the all-trans ferroelectric phase can be distinguished from the transmittance spectra, demonstrating that the ferroelectric phase of P(VDF–TrFE) is maintained in the nanocomposites.

### 3.2. Exceptionally High Negative ECEs of Nanocomposites Containing BT Nanowires

**Figure 2:**
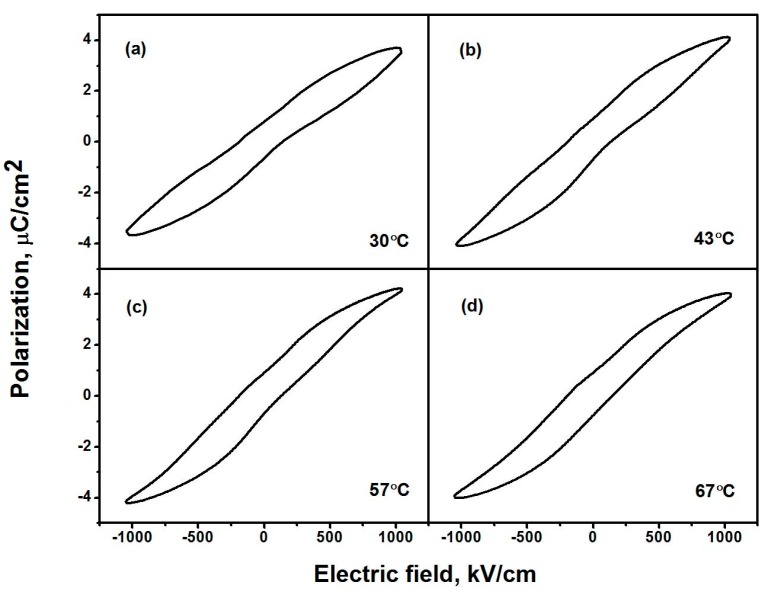
Hysteresis loops of P(VDF–TrFE)–BTNW nanocomposites with 5% volume fraction of BT nanowires at different temperatures.

Figure 2 shows the P-E loops of P(VDF–TrFE)–BTNW nanocomposites at 30, 43, 57 and 67 °C. At the lowest temperature measured the antiferroelectric-like double hysteresis loops are observed (Figure 2a), which are different from those observed in typical antiferroelectric ceramics where the sub-loops are square-like. At a higher temperature the remanent polarization increases, and a typical ferroelectric hysteresis loop is evident (Figure 2d). The changes from the double loops to a typical ferroelectric hysteresis loop as well as the increase of remanent polarization are gradual when the temperature increases, suggesting a phase transition from anti-ferroelectric to ferroelectric states.

**Figure 3:**
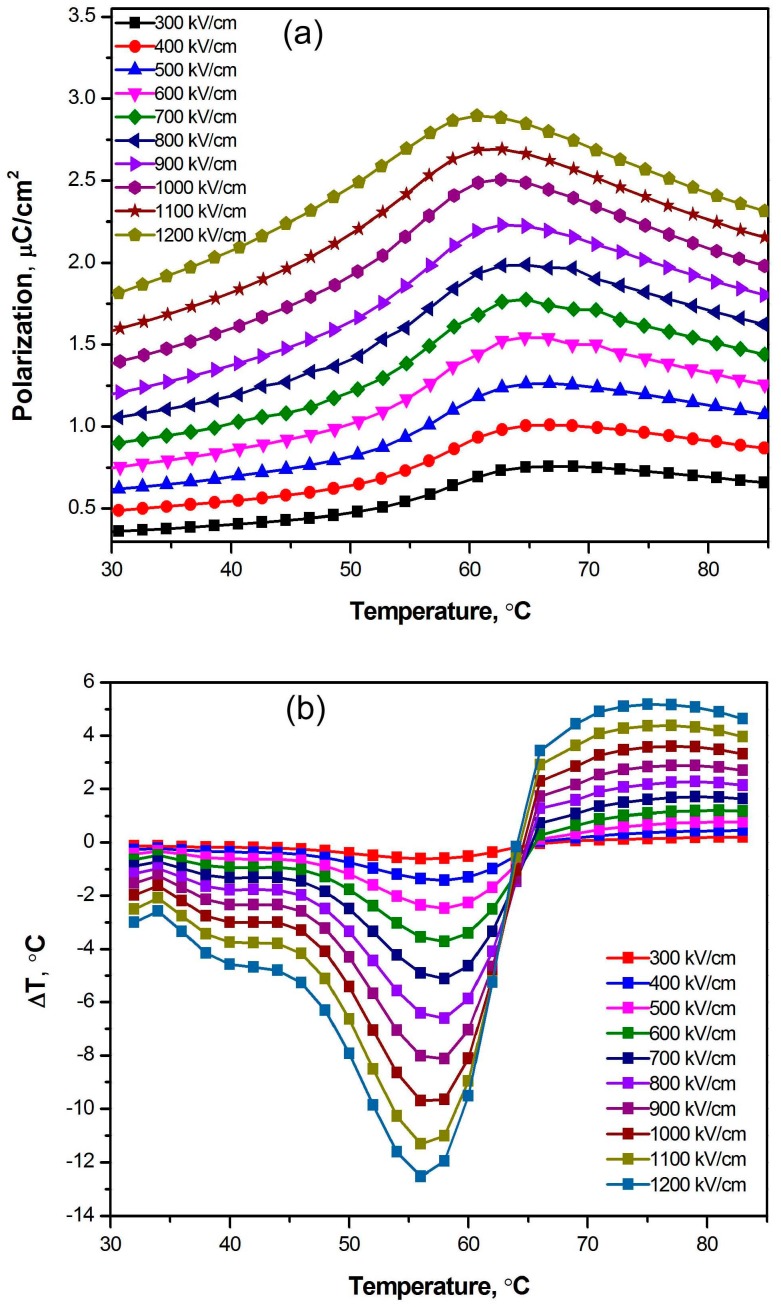
Temperature-dependent polarizations (**a**) and negative ECE temperature changes Δ*T* (**b**) in the P(VDF–TrFE)–BTNW nanocomposites with 5% volume fraction of BT nanowires.

As shown in Figure 2, the temperature-dependent remanent polarization (0.76, 0.95, 0.93 and 0.98 μC/cm^2^) and coercive field (142, 138, 120 and 139 kV/cm) exhibit abnormal behavior, i.e., the remanent polarization increases with increasing temperature below 67 °C. Figure 3a shows the temperature-dependent polarizations under electric fields of 300–1200 kV/cm in the thick-film P(VDF–TrFE)–BTNW nanocomposite with 5% volume fraction of BT nanowires. As shown in Figure 3a, the polarization first exhibits an abnormal increase with increasing temperature, reaching a maximum at 60–70 °C. Then the polarization decreases as the temperature is further increased. The ECE temperature change *ΔT* is calculated by the thermodynamic Maxwell’s relation,

(1) $$\Delta T=-\frac{T}{\rho C}\int_{E1}^{E2}{\left(\frac{\partial D}{\partial T}\right)}_{E}dE,$$

where *ρ* is the density and *C* is specific heat capacity and *E*_1_ and *E*_2_ are the lower and upper limits of the applied fields, respectively. The conventional (positive) giant electro-caloric effect results from the large pyroelectric coefficient (∂D/∂T)_E_ or (∂P/∂T)_E_ which is determined by the decrease of polarization with increasing temperature. However, in the P(VDF–TrFE)–BT nanocomposites a negative electro-caloric effect at a temperature lower than 60 °C can be observed, which is attributed to the abnormal increase of polarization with increasing temperature. Figure 3b shows the negative electro-caloric effect of P(VDF–TrFE)–BTNW nanocomposite calculated by the thermodynamic Maxwell’s equation, which is about −12.5°C, much better than any other results reported to date [10,11,12,13,14,15,16,17].

In both positive and negative ECEs reported to date, usually, the ECE temperature changes of bulk and thick-film samples [3,4,5,6,7,8,9] are much smaller than their thin-film counterparts since the electric fields applied on the thin-film samples could be 2–10 times higher [11,12,13,14,15]. In this study, we also compare the ECEs of P(VDF–TrFE)–BTNW thin-film and thick-film samples with the same composition. It is found that the thin-film samples exhibit meaningful ferroelectric hysteresis behavior up to an applied filed of 4200 kV/cm, and their highest negative ECE temperature change and refrigeration effect calculated from Equation (1) are 13.5 °C and 8.3 J/g, respectively, comparable to those of the thick-film samples. Therefore, thick-film P(VDF–TrFE)–BTNW nanocomposites could be much more promising ECE refrigerants since they possess exceptionally high ECE, low cost in processing and have effectiveness in implementation of ECE cooling.

**Figure 4:**
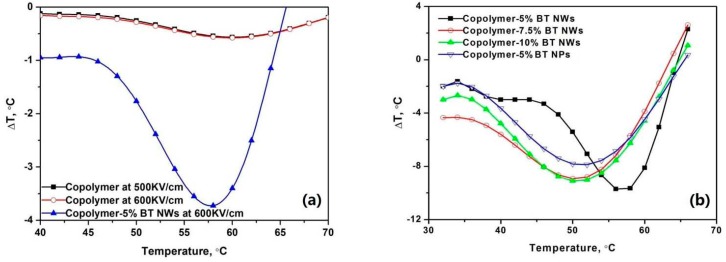
Comparisons on the negative ECEs in (**a**) pristine P(VDF–TrFE) copolymer and the P(VDF–TrFE)–BTNW nanocomposites; (**b**) P(VDF–TrFE)–BT nanocomposites containing BTNWs or BTNPs, under an electric field of 1 MV/cm.

Although the pristine P(VDF–TrFE) copolymer exhibits a negative ECE, its absolute value is rather small (<1 °C), and does not increase with increasing electric field in the range of 500 to 600 kV/cm. With the addition of BT nanowires, the absolute value of the negative ECE of nanocomposites increases significantly, by more than 3.8 °C under an electric field of 600 kV/cm, compared to that of pristine P(VDF–TrFE) copolymer. The negative ECEs of various nanocomposites under the same electric field of 1.0 MV/cm are shown in Figure 4. Compared to nanocomposites containing BT nanoparticles with the same volume fraction of 5%, the nanocomposites containing BT nanowires exhibit a stronger negative ECE. Among the nanocomposites investigated, the absolute value of negative ECE is found to be the largest in the nanocomposites containing 5% volume fraction of BT nanowires, while it slightly decreases with increasing content of BT nanowires. The nanocomposites containing BT nanoparticles exhibit a relatively weak negative ECE compared with those of nanocomposites containing BT nanowires.

It is argued that the antiferroelectric and relaxor features of La-doped PZT are responsible for the negative ECE [14]. In fact, negative ECE is observed in pristine P(VDF–TrFE) copolymer (Figure 4a) which has been reported to exhibit anti-ferroelectricity caused by the imperfections in its crystal structure [20]. Hence it is speculated that the negative ECE in the nanocomposite might be caused by its anti-ferroelectricity, as suggested by the anti-ferroelectric-like hysteresis loops shown in Figure 2. Nevertheless, the effects of nanofiller geometries and contents on the negative ECE in the nanocomposites cannot be well explained by their anti-ferroelectric-like properties. The mechanisms of negative ECE tuned by the geometries and contents of nanofillers are discussed in Section 4.

As shown in the SEM images (Figure 1c), BT nanowires are not straight and they are entangled with each other. Because the spontaneous polarization of BT nanowires is less affected by their diameters, the localized electric fields near the BT nanowires could be strong enough to alternate the polar states of the P(VDF–TrFE) matrix. Hence the entangled BT nanowires dispersed in P(VDF–TrFE) matrix could affect the ordered all-trans structure (TTTT) of P(VDF–TrFE) molecule chains due to the interaction between dipoles in the long chains of P(VDF–TrFE) and local electric fields near the BT nanowires. As a consequence, the dipole moments of the molecule chains could be modulated to form an anti-ferroelectric state. Although the exact crystal structure in the anti-ferroelectric state is not known, it might be a non-polar phase with some characteristics of both α and β phases, as demonstrated by the XRD results on the {110} planes (Figure S1).

## 4. Discussion

**Figure 5:**
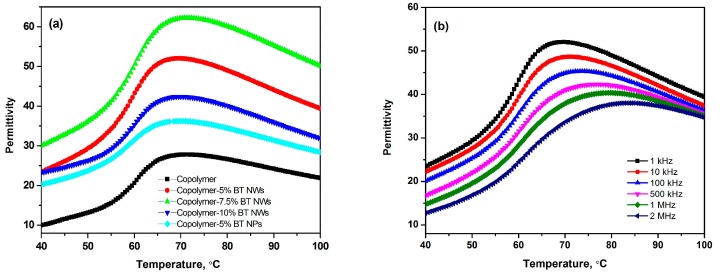
(**a**) The temperature-dependent permittivity of P(VDF–TrFE) copolymer and P(VDF–TrFE)–BT nanocomposites measured at 1 kHz. (**b**) The dielectric relaxation of P(VDF–TrFE)-BTNW nanocomposite containing 5% volume fraction of BT nanowires.

**Figure 6:**
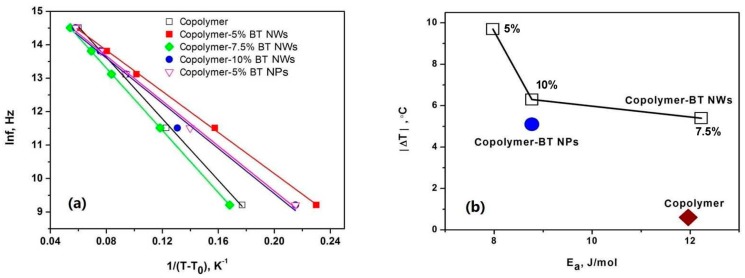
(**a**) Determination of the activation energies of dielectric relaxation in P(VDF–TrFE) and P(VDF–TrFE)–BT nanocomposites. (**b**) The relation between the negative ECE at 56 °C and the activation energy in P(VDF–TrFE) and P(VDF–TrFE)–BT nanocomposites.

Temperature-dependent dielectric constants at different frequencies are used to characterize the relaxor properties of P(VDF–TrFE) copolymer and P(VDF–TrFE)–BT nanocomposites. Figure 5 shows the temperature-dependent permittivity for P(VDF–TrFE) copolymer and P(VDF–TrFE)–BT nanocomposites at 1 kHz. With the addition of nanofillers, the permittivity of nanocomposite is improved compared to that of pristine P(VDF–TrFE) copolymer. Furthermore, the nanocomposites containing BT nanowires possess larger permittivity than those containing BT nanoparticles. For the nanocomposites containing BT nanowires, the permittivity first increases and then decreases with increasing volume fraction of BT nanowires and maximum permittivity occurs in the nanocomposites containing 7.5% volume fraction of BT nanowires. In the permittivity versus temperature curves a broad peak near 70 °C is attributed to the FE-to-PE transition with relaxor characteristics, which could be characterized by the Vogel–Fulcher–Tammann (VFT) relation as follows [21]:

(2) $$f=f_{0}e^{-E_{a}/k_{B}\left(T-T_{0}\right)},$$

where *f* is the test frequency and *T* is the peak temperature; *E*_a_ is the apparent activation energy, *f*_0_ is the attempt frequency and *T*_0_ is the freezing temperature. As shown in Figure 6a, the dielectric relaxation behaviors of the nanocomposites containing 5% volume fraction of BT nanowires are fairly consistent with the VFT relation and the freezing temperature *T*_0_ is determined to be 67.8 °C (or 341 K).

Based on the Kauzmann paradox for glassy solids, the entropy *S* of materials in a glass state relative to that (*S*_0_) of a fully ordered state can be written as: [22,23]

(3) $$\Delta S=\Delta S_{0}e^{-E_{a}/k_{B}\left(T-T_{K}\right)},$$

which could become zero at a finite temperature *T*_K_, the so-called Kauzmann temperature. Since the VFT relation is based on the Kauzmann theory for the dielectrics with relaxor characteristics, the Kauzmann temperature is equal to *T*_0_ = 67.8 °C. It can be found that *T*_K_ is very close to the critical temperature (64.8 °C) where the ECE changes from negative values to positive values, as shown in Figure 3. Such consistency suggests that the negative ECE in the nanocomposites might be related to their glassy polar states.

According to Equation (3), at *T*_K_ the change in configurational entropy Δ*S* = *S* − *S*_0_ becomes zero. While above *T*_K_, Δ*S* > 0, meaning that the configurational entropy *S* of an ordered states could be increased or decreased under the removal or application of an electric field, respectively. Therefore the conventional ECE (positive ECE) is exhibited. However, below *T*_K_, Δ*S* equals zero, meaning that *S* = *S*_0_ and *S* cannot be further reduced. Therefore, under the application of an electric field, *S* would always increase and the negative ECE occurs below *T*_K_.

The aforementioned mechanisms for the negative ECE also imply that the activation energy *E*_a_ of the dielectric relaxation process can be used to measure the strength of negative ECE. As shown in Figure 6a, the activation energies of P(VDF–TrFE) and the nanocomposites are determined by the VFT plots. Figure 6b shows the relation between *E*_a_ and the absolute value of negative ECE. It can be seen that the nanocomposite containing 5% volume fraction of BT nanowires has the smallest activation energy, meaning that the negative ECE more easily occurs in such nanocomposite compared with those in other nanocomposites. The absolute value of negative ECE increases with decreasing activation energy, which is consistent with that predicted by the mechanisms. 

**Figure 7:**
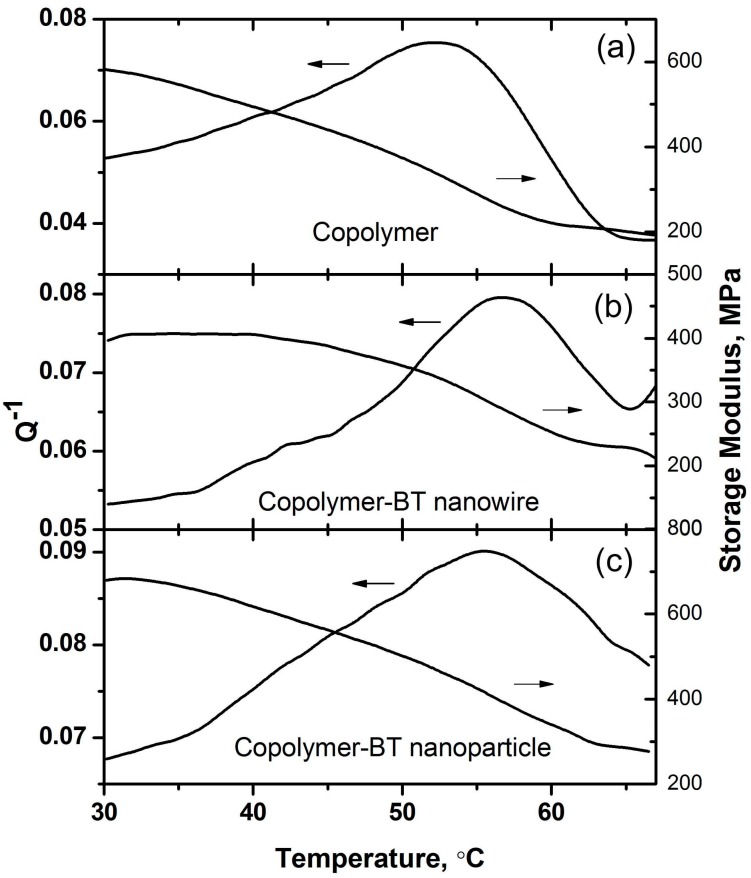
The effects of nanofiller geometries on the mechanical loss and storage modulus (at 1 Hz) of P(VDF–TrFE)–BT nanocomposites. (**a**) Copolymer; (**b**) Copolymer-5% BT nanowires; (**c**) Copolymer-5% BT nanoparticles.

Compared to the dielectric relaxation analysis, the dynamic mechanical analysis can be utilized to characterize the effects of nanofiller geometry on the structural phase transition accompanied by the transition from a ferroelectric state to a paraelectric disordered state. In temperature-dependent mechanical loss (Q^−1^), the phase transition can be identified by the peaks at different testing frequencies. Figure S3 shows the mechanical loss and storage modulus of pristine P(VDF–TrFE) and the P(VDF–TrFE)–BTNW nanocomposites, where the structural transition can be identified by a peak in the Q^−1^ curve. The addition of BT nanowires significantly shifts the Q^−1^ peak to a higher temperature under the same testing frequency, as shown in Figure 7. Compared with the effects of nanowires, the addition of BT nanoparticles with the same content shifts the Q^−1^ peak very slightly, as shown in Figure 7, indicating a relatively weak interfacial interaction during mechanical relaxation. The shift of Q^−1^ peak thus implies a strong interaction between the P(VDF–TrFE) and BT nanowires, and this leads to the enhanced negative ECE.

It is noted that the Q^−1^ peaks in the mechanical spectra for P(VDF–TrFE) and P(VDF–TrFE)–BT nanocomposites are of the relaxation type, which are different from those frequency-independent Q^−1^ peaks for a structural transformation usually observed in conventional ferroelectric materials [13,24]. These features of mechanical relaxation thus further demonstrate the existence of glassy polar states in the materials we investigated. These are closely related to their structural characteristics. Hence, the stronger interaction between the P(VDF–TrFE) and BT nanowires leads to more disordered structures in the nanocomposites containing 5% volume fraction of BT nanowires. As suggested by the Kauzmann theory, the nanocomposites containing 5% volume fraction of BT nanowires could exhibit better negative ECE performance than those containing BT nanoparticles and BT nanowires with different volume fractions.

## 5. Conclusions

In summary, BT nanowires and nanoparticles with typical sizes below 30 nm are synthesized via a one-step hydrothermal route for the fabrication of P(VDF–TrFE)–BT nanocomposites. A significantly enhanced dielectric property and ECEs are observed in these lead-free ferroelectric nanocomposites, compared with those of pristine P(VDF–TrFE). The effects of BT nanofillers on the nanocomposites are investigated by dielectric and mechanical relaxation analyses. The enhanced ECE is found to be related to the addition of BT nanowires with high specific areas and spontaneous polarizations. Significantly, a negative ECE larger than 12 °C is found in the nanocomposites consisting of P(VDF–TrFE) and 5% volume fraction of BT nanowires. This ECE is much larger than those of lead-containing ferroelectrics. The mechanisms of negative ECE and the enhanced negative ECE in the nanocomposites consisting of P(VDF–TrFE) and BT nanowires are explained by the Kauzmann theory on glassy polar states and the interaction between the nanofillers and the copolymer matrix. The nanocomposites with their enhanced negative ECE tuned by the geometries of BT nanofillers provide us with more options for small-sized ECE coolers for environmentally-friendly solid-state cooling technologies.

## Supplementary Materials

The following are available online at www.mdpi.com/2073-4360/9/8/315/s1, Figure S1: XRD results, Figure S2: FT-IR spectra, Figure S3: DMA results.
