# Supplementary material for: Exceptionally High Negative Electro-Caloric Effects of Poly(VDF–co–TrFE) Based Nanocomposites Tuned by the Geometries of Barium Titanate Nanofillers
Source: Polymers (Basel). 2017 Jul 28;9(8):315. doi: 10.3390/polym9080315 (PMC6418831; doi:10.3390/polym9080315)
Supplement: Supplementary file 1 [file polymers-09-00315-s001.pdf]

Supplementary Information

Structural, ferroelectric and electro-caloric properties of P(VDF-TrFE) based nanocomposites tuned by the geometries of barium titanate nanofillers

Zhi-Yuan Jiang[1], Guang-Ping Zheng[1,*], Xiu-Cheng Zheng[2,*], and Hao Wang[3,*]

[1] Department of Mechanical Engineering, Hong Kong Polytechnic University, Hung Hom, Kowloon, Hong Kong, China;

[2] College of Chemistry and Molecular Engineering, Zhengzhou University, Zhengzhou 450001, China;

[3] Institute of Nanosurface Science and Engineering, Shenzhen University, Shenzhen 518060, China;

* Correspondence: mmzheng@polyu.edu.hk; zhxch@zzu.edu.cn; whao@szu.edu.cn

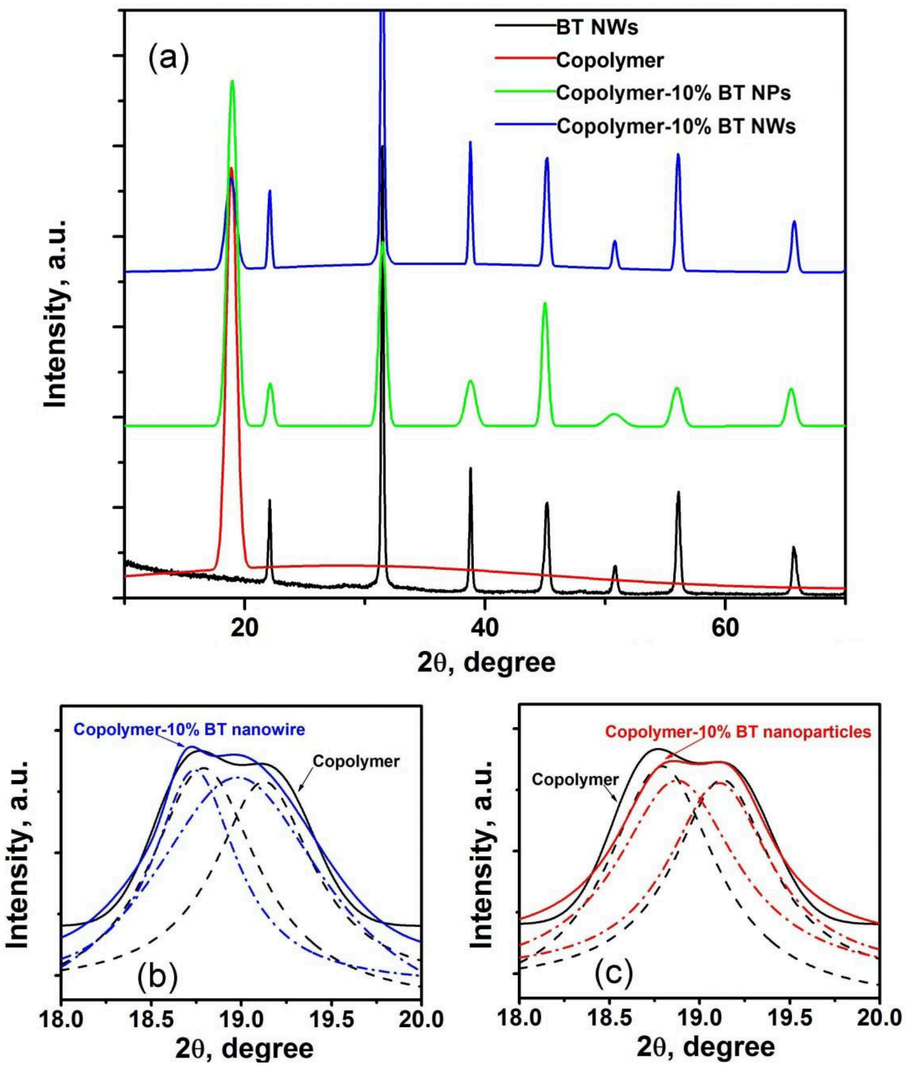

Figure S1. (**a**) The XRD patterns of BT nanowires, P(VDF-TrFE) and P(VDF-TrFE)-BT nanocomposites containing BT nanowires or BT nanoparticles. (**b**) The effects of BT nanowires on the (110) peaks of P(VDF-TrFE) matrix. (**c**) The effects of BT nanoparticles on the (110) peaks of P(VDF-TrFE) matrix.

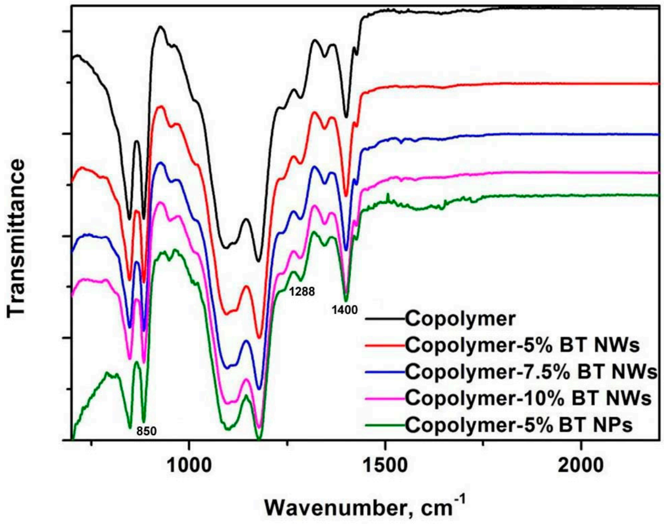

Figure S2. The FT-IR spectra of P(VDF-TrFE) and P(VDF-TrFE)-BT nanocomposites containing BT nanowires (NWs) or BT nanoparticles (NPs). The signals corresponding to the polar β phase in P(VDF-TrFE) are indicated.

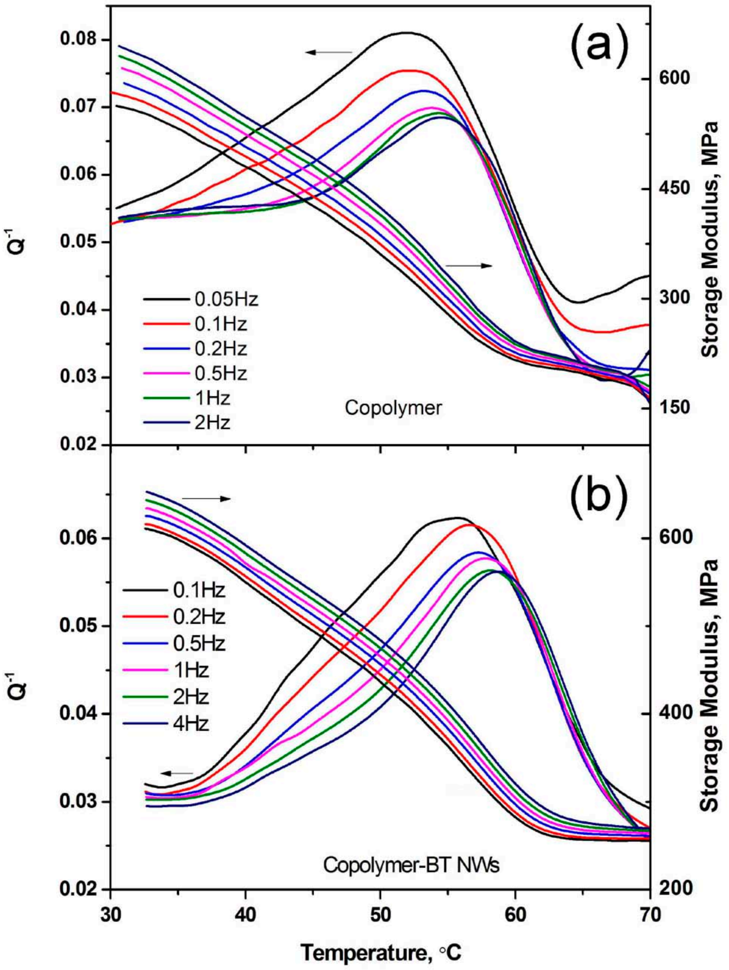

Figure S3. Mechanical loss (Q^{-1}) and storage modulus of P(VDF-TrFE) (a), and P(VDF-TrFE)-BTNW nanocomposite containing 5% volume fraction of BT nanowires (b).
